# New Appliances and Things Medical

**Published:** 1905-07-29

**Authors:** 


### NEW APPLIANCES AND THINGS MEDICAL.

[We shall be glad to receive, at our Office, 28 & 29 Southampton Street, Strand, London, W.C., from the manufacturers, specimens of all new preparations and appliances which may be brought out from time to time.]

### A NEW TRUSS.

(R. RAE, 1 WHARTON ROAD, WEST KENSINGTON, W.)

MR. RICHARD RAE has forwarded for our inspection a truss which in design is ingenious. The spring is formed out of a steel wire an eighth of an inch thick, which is tempered so as to be capable of adjustment by forcible bending or straightening without losing the qualities necessary in a truss spring. The truss is so designed that when it is in position the spring rests above the crest of the ilium, instead of passing round between the crest of the ilium and the great trochanter. If, as seems possible, this arrangement is found in practice to render a perineal strap unnecessary a great point will have been gained, although it appears from preliminary trials which we have made that the position of the spring to a slight extent hampers the patient's free movement, so that the abolition of the understrap will not be attained without a certain small compensatory disadvantage.

### THREE STAR SWISS DRY MILK.

(SWISS DRY MILK COMPANY, GLOCKENTHAL, NEAR THOUNE, SWITZERLAND).

THE preparation before us is a dry milk powder, obtained by the Just Hatmaker process, and produced in Switzerland. The milk, as to be consumed, is made from the powder by gradually adding hot—not boiling water. The milk thus prepared has a good flavour and corresponds to fresh milk in that it contains, per unit volume, the same solid constituents. The volume of the powder is so arranged that in order to make milk of average richness the contents of the tin must be treated with two and a half tins of warm water. The milk powder keeps and is naturally very portable and possesses the additional advantage of being sterile. Those unacquainted with the conditions of life amongst the poor in large cities do not grasp the enormous advantage of a preparation of milk that will keep. One of the most cogent arguments in favour of the use of preservatives in milk was the fact that although dairies, with due cleanliness and care, could supply milk in a perfectly sound condition without preservatives, yet, nevertheless, directly such milk was exposed to the conditions which obtain in the dwellings of the poor, it rapidly went sour, and as the purchasers could not afford to waste it it was drunk in this condition. Milk powders, such as the one before us, will keep under almost any conditions. This is an enormous point in their favour.

